# Budget impact analysis of a Lifestyle-integrated Functional Exercise (LiFE) program for older people in Germany: a Markov model based on data from the LiFE-is-LiFE trial

**DOI:** 10.1186/s12877-024-04802-y

**Published:** 2024-02-23

**Authors:** Judith Dams, Sophie Gottschalk, Michael Schwenk, Corinna Nerz, Clemens Becker, Jochen Klenk, Carl-Philipp Jansen, Hans-Helmut König

**Affiliations:** 1 Department of Health Economics and Health Services Research, University Medical Center Hamburg- Eppendorf, Hamburg Center for Health Economics, Hamburg, Germany; 2 Department of Sport Science, Human Performance Research Centre, University of Konstanz, Constance, Germany; 3 Department of Clinical Gerontology and Geriatric Rehabilitation, Robert Bosch Hospital, Stuttgart, Germany; 4 Institute of Epidemiology and Medical Biometry, Ulm University, Ulm, Germany; 5 IB University of Applied Health and Social Sciences, Study Centre Stuttgart, Stuttgart, Germany; 6 Center for Geriatric Medicine, University Hospital Heidelberg, Heidelberg, Germany

**Keywords:** Budget-impact analysis, Economic evaluation, Falls, Physical activity, Exercise

## Abstract

**Background:**

Fall prevention is important for healthy ageing, but the economic impact of fall prevention are scarcely investigated. A recent cost-effectiveness analysis compared a group-delivered Lifestyle-integrated Functional Exercise Program (gLiFE) with an individually-delivered program (LiFE) in community-dwelling people (aged ≥ 70 years) at risk of falling. In addition, the current study aimed to analyze the budget impact of LiFE and gLiFE, compared with standard care in Germany.

**Methods:**

A Markov model was developed to reflect falls and associated care needs for community-dwelling persons over 5 years. The intervention effects of LiFE and gLiFE were shown to be equivalent in a non-inferiority trial, although the costs differed. Outpatient, inpatient, and intervention costs were assessed from a payer’s perspective. The effect of parameter uncertainty was assessed in sensitivity analyses.

**Results:**

The budget impact due to intervention costs was €510 million for LiFE and €186 million for gLiFE. Over five years, health care expenditures were €35,008 million for those receiving standard care, €35,416 million for those receiving LiFE, and €35,091 million for persons receiving gLiFE. Thereby, LiFE and gLiFE could prevent 2700 deaths and 648,000 falls over 5 years. Parameter uncertainties in the risk of falling, uptake of an intervention offer, and in the intervention effects had a major influence; thus cost savings for LiFE and gLiFE compared with standard care could be achieved for individuals with a high risk of falling.

**Conclusions:**

The results revealed that cost savings for LiFE and gLiFE compared with standard care could only be achieved for individuals at high risk of falling, with gLiFE being superior to LiFE. Future research should consider benefits and aspects of fall prevention beyond falls (e.g., physical activity, social aspects, and personal preferences of participants).

**Trial registration:**

The study was preregistered under underclinicaltrials.gov (identifier: NCT03462654) on 12th March 2018; https://clinicaltrials.gov/ct2/show/NCT03462654.

**Supplementary Information:**

The online version contains supplementary material available at 10.1186/s12877-024-04802-y.

## Background

Falls are particularly common among older people. Statistically, one in three people over the age of 65 falls at least once in a year, with many experiencing more than one fall [1, 2]. After a fall, 75–90% of individuals require outpatient treatment for minor injuries, while 10–25% of falls result in major injuries requiring hospitalisation, such as fall-related fractures, head trauma or internal injuries [3, 4]. In addition to inpatient treatment in general hospitals, patients with major injuries often require long-term rehabilitation to restore mobility. Furthermore, the need for formal and informal care often increases after major injuries [5]. Consequently, falls are associated with a high economic burden of direct costs, ranging from purchasing power parity (US-$PPP) 2,044 to US-$ PPP 25,955, with inpatient costs being particularly high [6, 7].

In recent decades, effective prevention programs have been developed to reduce the risk of falling by improving balance and/or mobility. Thereby, fall-related consequences such as injuries are avoided, which in turn reduces health care costs [8, 9]. Fall prevention programs had often been found to be cost-effective, meaning that the additional intervention costs were justified by the effects gained [8, 9]. However, international results are rarely transferable to the German health care system and German economic evaluations are scarce. In Germany, recently published results from the LiFE-is-LiFE project found a group-delivered version of the Lifestyle-integrated Functional Exercise program (gLiFE) as well as the original, individually delivered version (LiFE), to increase walking activity and reduce the number of falls [10, 11]. The project’s cost-effectiveness analyses showed that gLiFE was likely to be cost-effective compared to LiFE from a payer’s perspective because gLiFE participants gained more steps per day than LiFE participants [11, 12]. Thus, health insurances may be induced to implement gLiFE nationwide in Germany.

To date, however, the economic consequences of a nationwide implementation of such a programme have not been studied. For prevention programs in particular, it is important to determine the budget impact: Fall prevention programs aim to prevent falls so that the relevant event of a fall occurs less frequently. Since fall-related healthcare costs are only incurred by some persons, whereas the intervention costs of a fall prevention program are incurred by all persons who participate in the program, regardless of whether they would have fallen or not, the intervention costs for one person must be justified by the reduction in healthcare costs and the effects achieved through the avoided falls for another person. Thus, a budget impact analysis provides information on the expected budget for a nationwide implementation of a fall prevention program, but also on the long-term health care costs avoided by its implementation [13].

The current study therefore aimed to determine the budget impact of implementing LiFE and gLiFE from a health payer’s perspective using a Markov model based on good practice principles for budget impact analysis over a five-year time horizon [14, 15]. This involved comparing the long-term health care costs of both prevention programs with standard care if no fall prevention program was implemented.

## Methods

### Interventions and initial population and intervention effects

The Markov model simulated the costs of using health care services and the effectiveness of fall prevention programs in individuals receiving either LiFE, gLiFE, or standard care over a five-year time horizon. The individual LiFE program consisted of seven home visits of approximately one hour each, during which a professional trainer taught balance and strength exercises and how to integrate them into daily activities. In gLiFE, the same exercises were taught by two professional trainers to groups of 8 to 12 persons in seven sessions of about two hours each. More information on the two fall prevention programs can be found elsewhere [16, 17].

Community-dwelling persons at risk of falling aged ≥ 70 years were eligible to participate in LiFE or gLiFE if they were able to walk at least 200 m without help [16, 18]. Thus only persons from the German general population aged ≥ 70 years with outpatient care needs and without inpatient care needs entered the model. The ability to walk at least 200 m independently was assumed to be reflected by the care degree (‘Pflegegrad’) of participants of the LiFE-is-LiFE trial [10, 12]. In Germany, there are five care degrees defined by the German statutory long-term care insurance, ranging from minor impairments of independence (care degree I) to severe impairments (care degree V). Data from the LiFE-is-LiFE trial showed that recruited persons were without care needs or were predominantly in care degrees I or II. Therefore, the target population assumed in the Markov model were persons without care needs or with care degree I to II. Furthermore, it was assumed that 13% would take up the offer to participate in LiFE and gLiFE [10].

### Markov model design

**Fig. 1:**
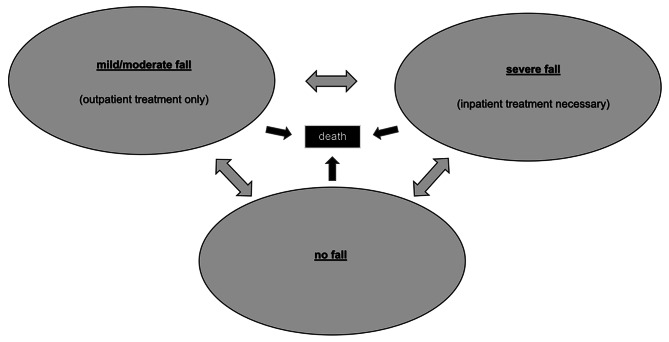
Markov model design with four Markov states: mild/moderate (outpatient treatment only) or severe fall (inpatient treatment necessary) or had not fallen in the previous year as well as death of persons

The design of the Markov model reflected the occurrence of falls through four Markov health states (Fig. 1). Individuals entering the model could either 1. have not fallen in the previous year, 2. have fallen and required outpatient treatment only (mild/moderate fall), 3. have fallen and required inpatient treatment (severe fall), or 4. have died [19]. During the simulation, persons could move between Markov states, except for the absorbing ‘death’ state.

### Transitions between health states

Transitions between Markov states were implemented using one-year cycles. Transition probabilities to experience a mild/moderate or severe fall were taken from the literature, according to which 26% of women fell within one year, of whom 9% had a severe fall requiring hospitalization [20, 21]. In comparison, men fell less frequently (16%) and had less severe falls (7%).

The Markov model further accounted for the probability of dying in each cycle, taken from age- and gender-specific mortality rates published by the German Statistical Office [22], taking into account the relative risk of dying from a fall [23].

In addition to age- and gender-specific transition probabilities, individuals in each Markov state (except death) were distinguished by the five care degrees [5]. Changes in the care degree were calculated using data from the German long-term care insurance [26, 27]. The care degree depended on the severity of the fall, i.e. more outpatient and especially more inpatient care was required for persons who experienced a severe fall [28], whereas no change in care needs was assumed for persons who experienced a mild/moderate fall compared to persons who did not fall. In the absence of data on the direct impact of a severe fall on the care degree, changes in the care needs (expressed as daily care times) following a hip fracture were used, as hip fractures are often the result of a severe fall. Daily care times of 0.75 or 2 h were assigned to care degree I-III, whereas care times of ≥ 4 h were assigned to care degree IV and V [5]. Thus, compared with persons who did not fall and those with a mild/moderate fall, 14% of the persons in care degree I-III and 9% in care degree IV with a severe fall were assumed to move up one care degree (Additional file 1).

### Intervention effects

Because the intervention effects were not significantly different between LiFE and gLiFE, as shown in the non-inferiority trial [17], the transition probabilities for the risk of falling were assumed to be reduced by 31% in persons who participated in either fall prevention program compared with standard care [10, 18].

### Fall-related and intervention costs

Data on fall-related and intervention costs were taken from the LiFE-is-LiFE trial [10, 18]. Fall-related costs from a payer’s perspective included outpatient physician services, general hospitalizations, and rehabilitation services. These were based on the health-related resource utilization of the LiFE-is-LiFE participants, which was monetarily valued in euros (€) based on standardized unit costs [29] and inflated to the year 2018 according to the consumer price index [30]. As a result, inpatient costs were lowest for persons who had not fallen, at €2310, while those with a mild/moderate or severe fall had inpatient costs of €2492 and €9483, respectively. Outpatient costs for physician services (€894) and rehabilitation (€44) were similar for persons who had fallen or not.

In addition to these costs, the intervention costs per person for the LiFE (€332) and gLiFE (€121) programs were included in the model based on data from the LiFE-is-LiFE trial [10].

Apart from the trial data, care costs by care degree were obtained from the German long-term care insurance [31]. According to these data, persons in care degrees I, II, III, IV, and V received an average annual benefit of €1128, €8770, €13,798, €16,780, and €20,116 for outpatient care, respectively. No benefits were paid for inpatient care for persons in care degree I, while persons in care degree II, III, IV, and V received €9249, €15,144, €21,300, and €24,060, respectively.

### Sensitivity analysis

Deterministic sensitivity analyses were used to vary several model parameters in order to investigate the effect of parameter uncertainty on the results. The initial population varied in age between 65 and 75 years and care degree (± 1). Regarding transition probabilities, mortality varied by ± 20% and the fall rate by ± 10%. In addition, the probability of a severe fall and the increase in costs due to a severe fall varied by ± 5%. The cost ranges for the intervention, inpatient, and outpatient services were ± 20% each. On the intervention side, both the intervention effect and the uptake rate of the intervention varied by ± 10%. In addition, the effect duration of the interventions varied between 1 and 5 years. Furthermore, subgroup analyses of a participation offer for people with different care degrees were conducted. In addition, parameter thresholds were identified for which cost savings could be achieved after 5 years. Multivariate sensitivity analyses were used to examine the relationship between fall rate and intervention costs and intervention effects. Probabilistic sensitivity analysis was performed with *n* = 10,000 iterations. Intervention effects were assumed to be beta-distributed, outpatient and inpatient costs were assumed to be gamma-distributed, and formal care costs were assumed to be normally distributed.

All parameter values used in the base case and sensitivity analyses and their sources are shown in Table 1. All statistical analyses were performed in Excel 2016 [Microsoft Corporation 2016. Microsoft Excel 2016. Redmond, WA: Microsoft Corporation].

**Table 1:** Base-case and sensitivity analyses parameter values

									Range for Sensitivity Analyses							
Variable				Base-case					Minimum			Maximum				Reference
Transition probability (%)																
		mortality		Mortality tables					± 20							[22]
		fall rate (♀/♂) age 65–69 70–74 75–79 ≥ 80		♀ 24 26 28 42		♂ 14 16 21 22			♀ 4 6 8 2	♂ 0 0 1 2		♀ 44 46 48 62		♂ 34 36 41 42		[28]
		severe fall rate (♀/♂)		9		7			4	2		14		12		[21]
		care after severe fall care degree I-III care degree IV-V		14 9					9 4			19 14				[5]
Costs (€, 2018)																
		care (out-/inpatient) care degree I care degree II care degree III care degree IV care degree V		outpatient 1128 8770 13,798 16,780 20,116	inpatient 0 9249 15,144 21,300 24,060				outpatient 0 3792 6540 8736 10,812	inpatient 0 7399 12,155 17,040 19,248		outpatient 2256 13,748 21,056 24,824 29,420	inpatient 0 11,098 18,172 25,560 28,872			[26]
		general hospitals without fall mild/moderate fall severe fall		2134 2134 6436					1707 1707 5148			2561 2561 7723				[10, 12]
		inpatient rehabilitation without fall mild/moderate fall severe fall		176 358 3047					141 286 2438			211 430 3656				[10, 12]
		outpatient rehabilitation		44					35			53				[10, 12]
		outpatient treatment		804					643			965				[10, 12]
		intervention gLiFE LiFE		121 332					266 97		398 145					[10, 12]
Intervention (%)																
		risk reduction of falls		31					21			41				[18]
		effect duration (years)		5					1			5				[18]
		uptake rate		13					3			23				[10, 12]
Initial population																
		age (years)		70					65			5				[17]
		care degree		≤ 2					≤ 1			≤ 3				[17]

♀: female /♂: male

LiFE: individual-based Lifestyle integrated Functional Exercise program; gLiFE: group individual-based Lifestyle integrated Functional Exercise program

## Results

### Cohort characteristics

At the beginning of the simulation, the cohort included *n* = 675,691 men and *n* = 879,722 women. Over a 5-year time horizon, *n* = 376,514 and *n* = 373,882 persons receiving standard care and fall prevention, respectively, died. Among those who survived, *n* = 2,094,481 falls (standard care) and *n* = 1,446,421 falls (fall prevention) occurred over 5 years. Assuming equal intervention effects for LiFE and gLiFE, as suggested by the data from the non-inferiority LiFE-is-LiFE trial, both fall prevention programs prevented 648,060 falls compared to standard care, including 517,017 falls among persons without care needs; 122,804 and 11,012 falls among persons with outpatient and inpatient care needs respectively.

### Base-case analysis

The budget impact due to intervention costs was €510 million for LiFE and €186 million for gLiFE. Over a 5-year time horizon, total health care costs including intervention costs were €35,008 million for persons receiving standard care, €35,416 million for persons receiving LiFE, and €35,091 million for persons receiving gLiFE (Table 2). This resulted in a cost saving of €325 million for gLiFE compared to LiFE. Both gLiFE and LiFE exceeded the cost of standard care by €83 million and €408 million, respectively. Formal care and inpatient treatment in general hospitals accounted for the largest proportion of health care costs, each accounting for 40% of total costs for persons receiving gLiFE, LiFE or standard care. Only 15% of health care expenditure was spent on outpatient treatment and 5% on inpatient or outpatient rehabilitation.

**Table 2:** Base-case results (€ in million, 2018)

	time horizon (years)						Total costs (over 5 years)		
cost categories	intervention phase	1st	2^sd^	3rd	4th	5th			
intervention cost									
LiFE	510								
gLiFE	186								
formal care standard care LiFE/gLiFE		1792 1793	2328 2331	2799 2805	3198 3208	3527 3541		**13,644** **13,679**	
outpatient treatment standard care LiFE/gLiFE		1183 1183	1125 1126	1064 1065	1000 1002	934 936		**5305** **5311**	
outpatient rehabilitation standard care LiFE/gLiFE		65 65	62 62	58 58	55 55	51 51		**290** **291**	
general hospitals standard care LiFE/gLiFE		3158 3153	3005 3001	2843 2840	2670 2670	2495 2496		**14,170** **14,160**	
inpatient rehabilitation standard care LiFE/gLiFE		353 324	338 310	321 294	302 277	285 260		**1598** **1465**	
**Cumulative total costs for the previous 1 to 5 years**									
**standard care**		**6550**	**13,407**	**20,492**	**27,716**	**35,008**			
**LiFE**		**7029**	**13,858**	**20,921**	**28,132**	**35,416**			
**gLiFE**		**6704**	**13,533**	**20,596**	**27,807**	**35,091**			

### Sensitivity analyses

**Fig. 2:**
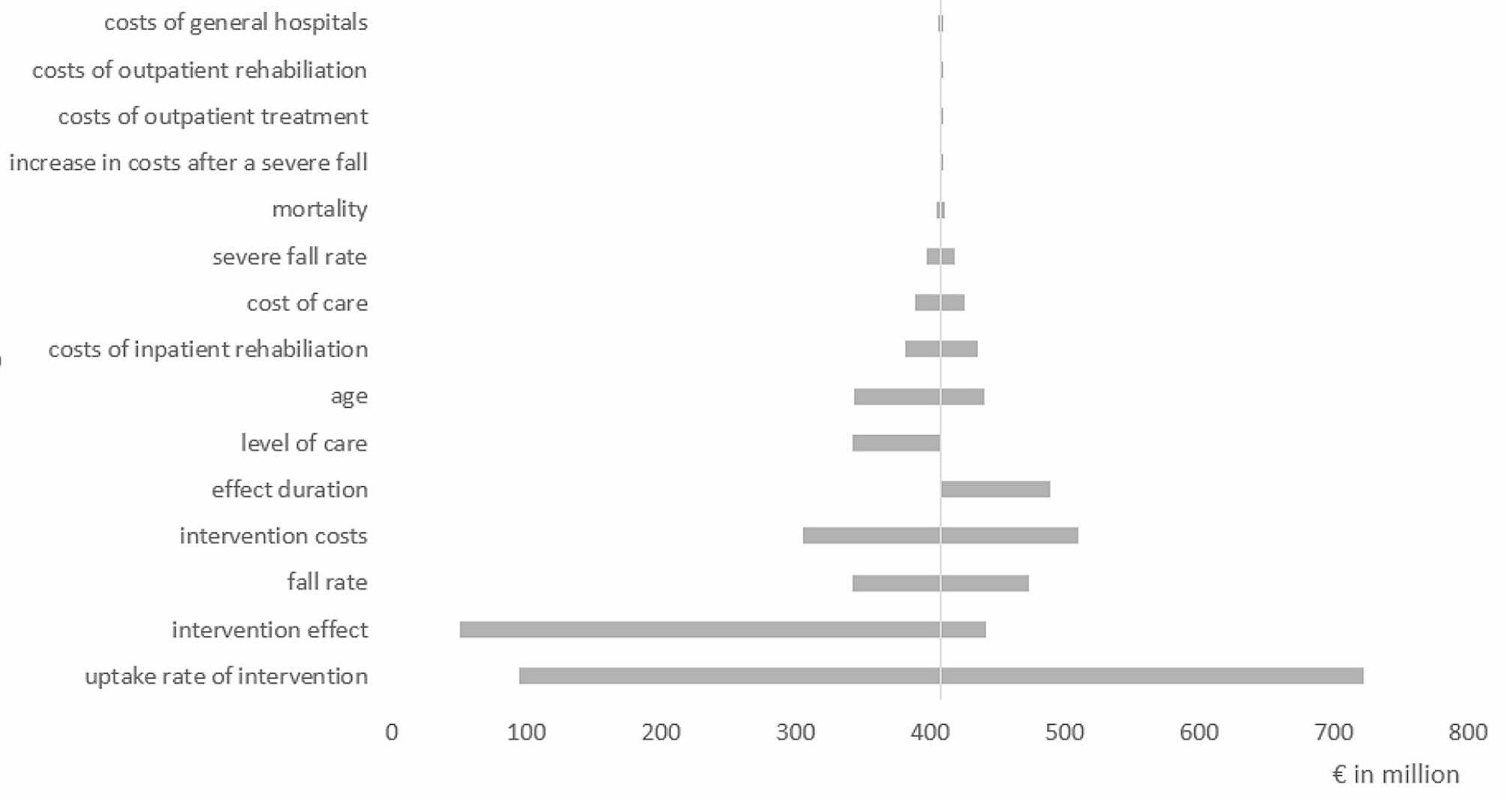
Tornado-diagram: cost differences for LiFE compared with standard care after 5 years

**Fig. 3:**
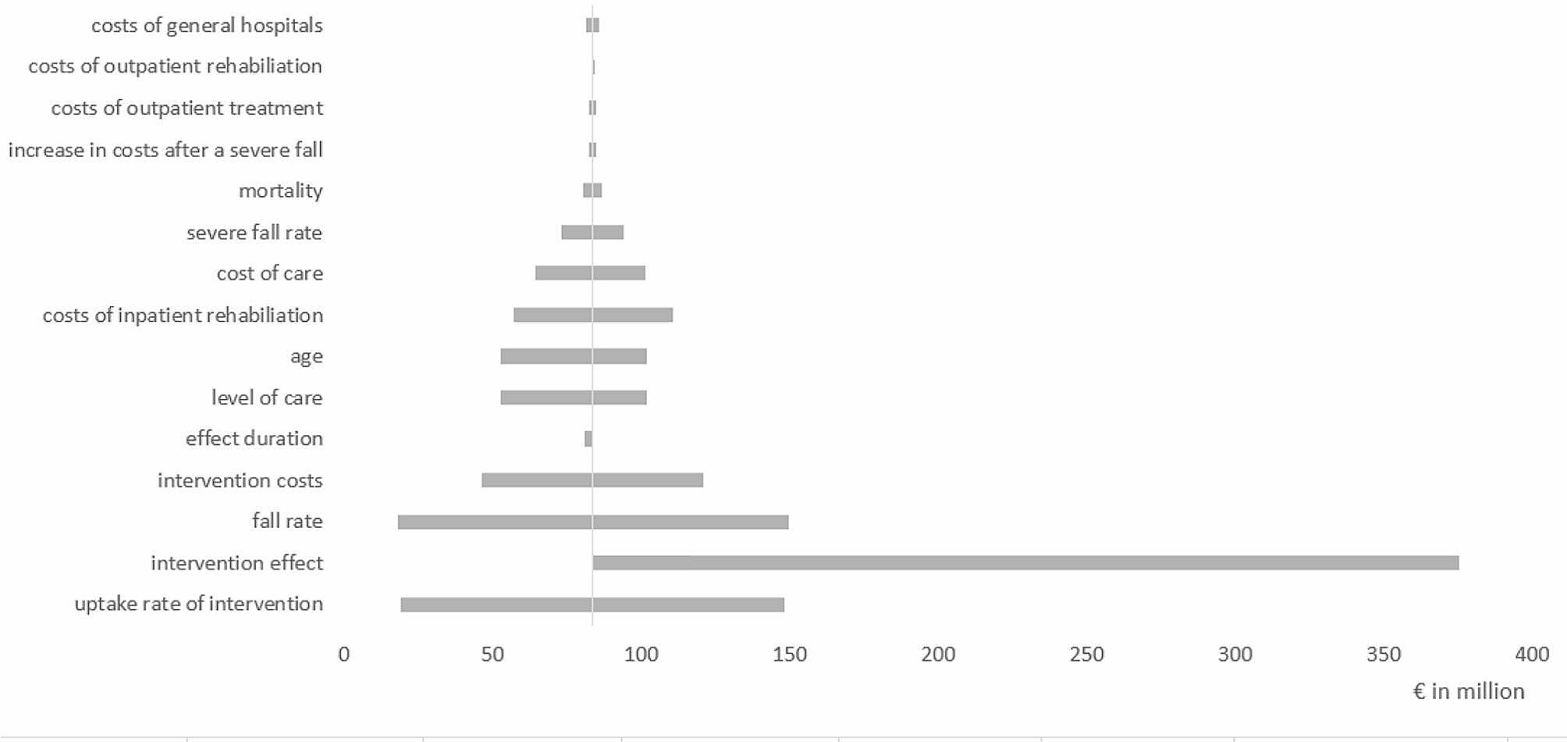
Tornado-diagram: cost differences for gLiFE compared with standard care after 5 years

Sensitivity analyses showed cost savings for gLiFE compared to LiFE ranging from €75 million to €574 million over 5 years (data not shown). In contrast, neither gLiFE nor LiFE showed any cost savings compared to standard care (Figs. 2 and 3). The parameters that most influenced the result in terms of potential cost savings were the cost and effectiveness of the intervention, the uptake rate of the intervention, and the rate of falls in the population. Subgroup analyses, in which groups of people with different care degrees were allowed to participate in the interventions, showed that the intervention tended to achieve higher cost savings for people with higher care degrees. In particular, there were high cost savings for formal care, outpatient treatment, and inpatient rehabilitation. For example, cost savings within in the 5-year period for gLiFE or LiFE compared with standard care could only be achieved if the intervention effect was increased to a fall reduction of 57%, if only persons with at least a 57% annual risk of falling were allowed to participate in the intervention, or if the intervention costs were reduced to €66 per person.

Multivariate sensitivity analyses showed that reducing the intervention cost to €100, €80, €60, €40 or €20 would result in cost savings at 5 years if the annual risk of falling was 48%, 38%, 28%, 19% or 10%, respectively.

Probabilistic sensitivity analyses showed that in 19% of cases, costs were higher for persons receiving LiFE than for persons receiving standard care. When gLiFE was considered, costs were higher than cost for standard care in 12% of the cases (Additional file 2).

## Discussion

The economic feature of prevention programs is that money is invested before the actual event (e.g., a fall) occurs, and it is not certain that this event would ever occur without the intervention. It is therefore important to determine the budget impact of such prevention programs before they are introduced nationwide. In the absence of economic data comparing gLiFE and LiFE with standard care, previous economic evaluations examined the cost-effectiveness of gLiFE compared with LiFE [11, 12]. The results showed that the cost-effectiveness of gLiFE was uncertain in terms of health-related quality of life or falls prevented at 6 and 12 months compared with LiFE. It remained unclear whether the intervention costs could be offset by a reduction in the fall-related follow-up costs over a period of more than 12 months, and what costs would be incurred if the programs were implemented on a large scale. Therefore, the current budget impact analyses modelled the long-term effects of a national implementation over 5 years. The base case analysis showed a budget impact of €186 million and €510 million, respectively, if either gLiFE or LiFE were implemented and delivered nationwide in Germany to prevent falls, resulting in additional costs of €325 million for LiFE compared to gLiFE. The incremental total costs due to health care utilization and intervention costs between gLiFE or LiFE and standard care were €83 million and €408 million, respectively. At the same time, it was found that a nationwide implementation of both programs would prevent 2,692 deaths and 648,060 falls, with an additional cost of €128 for gLiFE and €630 for LiFE for each fall prevented compared with standard care. The costs per death avoided were €30,832 and €151,560, respectively. Multivariate sensitivity analyses showed that cost savings would be possible if 1. only people with an increased risk of falling were offered participation, 2. higher intervention effects were achieved, or 3. the intervention costs were reduced.

Previous budget impact analyses have focused on interventions for people with osteoporosis to prevent falls [32] or fractures [33, 34], both of which are known to be associated with high costs, particularly for inpatient treatment and care [6]. The budget impact of the interventions offered ranged from US$26 million [34] to £2.70 billion [32], with cost savings possible for all interventions offered. The lifetime cost savings were up to £522 million [33], although a shorter time horizon of 5 years resulted in lower cost savings of £420 million [32]. In particular, cost savings were achieved by preventing hospitalization and nursing care [32, 33]. Therefore, combined with the results of the current sensitivity analyses, it is reasonable to expect that cost savings over standard care can also be achieved for LiFE and gLiFE by focusing on groups of people with an increased risk of falling. As persons with an increased risk of falling are likely to benefit more from fall prevention programs (i.e. achieve a higher intervention effect), targeting only this group of people could in turn lead to an additional positive effect on health care costs, as shown in the sensitivity analyses.

Furthermore, higher intervention effects for LiFE and gLiFE might be expected if the programs were offered more than once or if beside prevented falls further effects of both programs were taken into account. In the LiFE-is-LiFE trial, 6- and 12-month follow-up data showed improvements in the number of falls and, in particular, improvements in physical activity compared with baseline in both intervention groups [10, 11]. In addition to clinical intervention effects such as falls or physical activity, both program versions may have effects on a social level, as well as the well-being of participants. gLiFE promotes interaction with other participants through group exercises, while LiFE is characterized by individual support from the trainer. However, considering both physical activity and social effects in budget impact analyses is challenging because data on these aspects are not available for the general population, or are difficult to quantify (e.g., comprehensive assessment of the social effects of the interventions).

Ultimately, the impact on the budget always depends on the intervention cost. For example, in the case of fall prevention, reducing intervention costs can lead to a cost saving, e.g., reducing intervention costs for LiFE and gLiFE to €66 led to cost savings after 5 years. A reduction in intervention costs from the payer’s perspective could be achieved, for example, by introducing a co-payment for participants equal to the remaining intervention costs (€266 for LiFE and €55 for gLiFE). Indeed, a survey of participants’ willingness to pay in the LiFE-is-LiFE trial showed that the mean willingness to pay in the gLiFE group of €196 could well cover these co-payments, whereas the mean willingness to pay in the LiFE group of €228 would not [35]. However, a co-payment for participants could also reduce the uptake rate of the intervention, which would negatively affect the cost differences between fall prevention programs and standard care.

### From research to practice

The results showed that from a payer’s perspective, both fall prevention programs could, under certain conditions, lead to cost savings after 5 years compared to standard care. Severe falls in particular had an economic impact. However, severe falls are rare, so that intervention costs can only be offset if people at high risk of falling participate or if the uptake rate of an intervention is increased. It is also conceivable that the group size in the gLiFE intervention could be increased, so that this would have a cost-reducing effect on the cost of the intervention for an individual participant. The latter is characterised by a high degree of practicability, although it is important to ensure that the content is still readily understandable and the quality of exercises can be maintained despite the larger group size.

The decision to participate in a fall prevention program may depend on aspects other than fall prevention, such as social interaction or the accessibility of the location of the intervention (e.g., in sports clubs or community centers close to home). The promotion of social interaction would be the case, for example, with social prescribing, where social activities could be prescribed by the doctor or therapist for existing medical reasons (e.g. mental health conditions). This in turn could also have an effect on the health service utilisation and its costs. Whether this would actually increase uptake of the intervention or even effect costs in the long run is not known and should be the aim of future research. Therefore, reimbursement decisions for a fall prevention program should be based on outcomes such as mortality and social aspects in addition to the number of falls prevented. Furthermore, similar to other prevention programs in Germany, cost sharing between payers and participants would reduce costs for health and long-term care insurers and therefore might increase the likelihood of achieving cost savings.

### Strengths and limitations

To our knowledge, the present analysis is the first to assess the budget impact of a fall prevention program in Germany. It is also the first economic analysis to compare an individual and group fall prevention program with standard care. Both mortality and severity of falls were considered based on outpatient and inpatient care degrees, so that all costs due to falls were captured in the model. In addition, the model was able to represent the German general population based on gender- and age-specific secondary data from the Federal Statistical Office and long-term care insurance, so that most parameters were reliable and the results can be generalized for nationwide implementation.

One of the limitations is certainly that reliable parameter estimation was not possible for all parameters. First, there was a lack of data on the long-term effects of the two interventions. Sensitivity analyses showed that cost savings could be achieved after 5 years for an intervention cost of €66, whereas this was possible after 1 year for an intervention cost of €21. This means that intervention costs would have to be reduced by €55 to achieve cost savings if the effect of the intervention lasted only 1 year instead of 5 years. Therefore, we emphasize that both programs should be economically reevaluated based on long-term data after 5 years. Second, no data comparing gLiFE with standard care is available to date. Therefore, the effects between gLiFE and standard care were assumed to be equal to those of LiFE, as no significant differences in fall reduction were observed between LiFE and gLiFE [10]. Third, cost data for outpatient physician services and inpatient hospitalizations are uncertain because only *n* = 38 subjects were hospitalized in the 6 months prior to the baseline assessment of the LiFE-is-LiFE trial and only *n* = 110 persons had fallen [10]. However, sensitivity analyses did not find a significant effect of uncertainties in outpatient physician services and inpatient hospitalization costs on the results. Finally, multiple falls were not included in the model because the data on risk factors and numbers are difficult to generalize to the German general population.

## Conclusions

The current budget impact analysis found that LiFE and gLiFE only resulted in cost savings compared with standard care for individuals at high risk of falling, with gLiFE being superior to LiFE. Multivariate sensitivity analyses showed that cost savings were possible if the offer to participate was made to persons at high risk of falling, if a higher intervention effect was achieved or if intervention costs were reduced.

### Electronic supplementary material

Below is the link to the electronic supplementary material.

Supplementary Material 1

Supplementary Material 2

## Data Availability

The datasets generated and/or analyzed during the current study are not publicly available due to ethical and confidentiality concerns but are available from the corresponding author on reasonable request.

## Abbreviations

gLiFE: group-delivered Lifestyle-integrated Functional Exercise Program
LiFE: Lifestyle-integrated Functional Exercise Program
PPP: Purchasing Power Parity
