# Supplementary material for: Budget impact analysis of a Lifestyle-integrated Functional Exercise (LiFE) program for older people in Germany: a Markov model based on data from the LiFE-is-LiFE trial
Source: BMC Geriatr. 2024 Feb 23;24:186. doi: 10.1186/s12877-024-04802-y (PMC10893703; doi:10.1186/s12877-024-04802-y)
Supplement: Supplementary file 1 — Supplementary Material 1 [file 12877_2024_4802_MOESM1_ESM.docx]

**Additional file 1**

|  | | **persons who had a mild/moderate fall or had not fallen** | | | | | | **persons who had a severe fall** | | | | | |  |
| --- | --- | --- | --- | --- | --- | --- | --- | --- | --- | --- | --- | --- | --- | --- |
| transition… | | … to care degree | | | | | | … to care degree | | | | | |  |
|  |  | no care | I | II | III | IV | V | no care | I | II | III | IV | V |  |
| … from care degree | I | 0.012 | 0.676 | 0.237 | 0.062 | 0.012 | 0 | 0.012 | 0.536 | 0.377 | 0.062 | 0.012 | 0 |  |
|  | II | 0 | 0 | 0.803 | 0.144 | 0.039 | 0.013 | 0 | 0 | 0.663 | 0.284 | 0.039 | 0.013 |  |
|  | III | 0 | 0 | 0 | 0.813 | 0.144 | 0.043 | 0 | 0 | 0 | 0.723 | 0.234 | 0.043 |  |
|  | IV | 0 | 0 | 0 | 0 | 0.831 | 0.169 | 0 | 0 | 0 | 0 | 0.741 | 0.259 |  |
|  | V | 0 | 0 | 0 | 0 | 0 | 1 | 0 | 0 | 0 | 0 | 0 | 1 |  |

Transition probabilities for care degree in persons with a no/mild/moderate and severe falls.
