# Supplementary material for: Budget impact analysis of a Lifestyle-integrated Functional Exercise (LiFE) program for older people in Germany: a Markov model based on data from the LiFE-is-LiFE trial
Source: BMC Geriatr. 2024 Feb 23;24:186. doi: 10.1186/s12877-024-04802-y (PMC10893703; doi:10.1186/s12877-024-04802-y)
Supplement: Supplementary file 2 — Supplementary Material 2 [file 12877_2024_4802_MOESM2_ESM.docx]

Additional file 2

Results of probabilistic sensitivity analysis


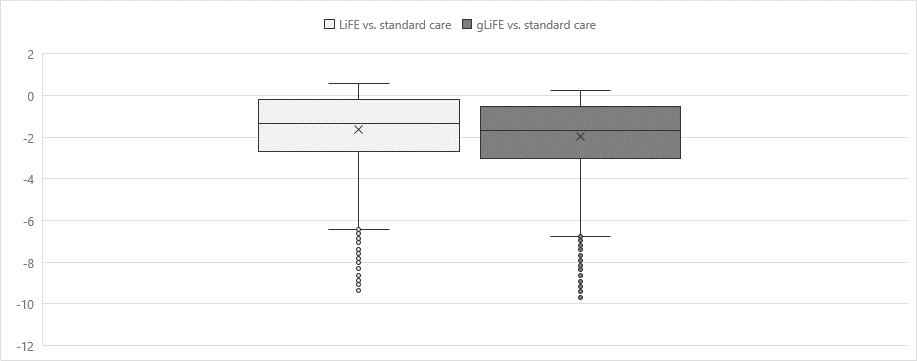


Differences in costs in billions of € between LiFE and standard care as well as between gLiFE and standard care, determined by probabilistic sensitivity analysis (n=10,000 iterations).
